# Optimizing adaptive neuro-fuzzy inference system model based Chaotic Harris Hawks algorithm for stock prediction

**DOI:** 10.1038/s41598-025-15022-8

**Published:** 2025-09-15

**Authors:** Zahraa Elsayed Mohamed, Ahmed Refaie Ali, Walid Dabour

**Affiliations:** 1 Department of Mathematics, Faculty of Science, Zagazig University, P.O. Box 44519, Zagazig, Egypt; 2 Department of Mathematics and Computer Science, Faculty of Science, Menoufia University, Shebin El Kom 32511, Menofia, Egypt

**Keywords:** AI, Harris Hawks algorithm, Anfis, Chaotic, Stock prediction, Neuroscience, Information technology, Scientific data, Software, Statistics

## Abstract

Artificial intelligence (AI) has become increasingly prevalent in the financial sector, particularly for predicting stock market movements. Due to the efficient market hypothesis, forecasting stock prices remains a challenging task, as prices are primarily influenced by fluctuating supply and demand. This study introduces an optimized Adaptive Neuro-Fuzzy Inference System (ANFIS) integrated with a Chaotic Harris Hawks Optimization (ChHHO) algorithm to enhance the accuracy of stock price predictions. The ChHHO method improves search space exploration and mitigates the risk of convergence to local optima, thereby enhancing predictive performance. This study is motivated by the need to improve the precision of stock price predictions, which are often hindered by the non-linear and chaotic nature of financial data. By combining ANFIS with the chaotic Harris Hawks Optimization, we aim to develop a model that addresses these challenges effectively. The proposed ANFIS-ChHHO model is evaluated on EGX index stock data using metrics such as Root Mean Square Error (RMSE), Mean Absolute Error (MAE), Standard Deviation (SD), and Theil’s U. Results demonstrate that the ANFIS-ChHHO model outperforms traditional methods in prediction accuracy.

## Introduction

A stock market is a financial exchange where stocks and other securities are traded. It serves as a platform where traders and investors buy and sell equities through designated trading venues known as stock exchanges. In recent years, artificial intelligence (AI) has gained significant traction in the financial sector, particularly in stock market applications.

AI algorithms can analyze vast datasets and utilize the results to support decision-making processes. This capability enhances the accuracy of investment decisions, trend identification, and stock price forecasting. By examining historical stock prices, corporate financial statements, and market trends, AI methods can provide valuable insights into future performance. Furthermore, AI systems can monitor real-time market conditions, detect potential opportunities, and respond dynamically to changes in the trading environment^1,2^.

In recent years, predicting stock market prices has become increasingly complex due to the inherent volatility and non-linear patterns. Advanced hybrid models, integrating machine learning with optimization algorithms, offer promising solutions. Among these, the combination of ANFIS and chaotic optimization techniques stands out. This study introduces a novel model that leverages the strengths of chaotic Harris Hawks Optimization to enhance the adaptive capabilities of ANFIS, thereby addressing the challenges of accuracy and stability in stock price forecasting.

Recent research has increasingly focused on employing deep learning and AI-based decision-making systems to enhance the accuracy of stock market predictions. Several studies have explored various models and techniques, each aiming to improve forecasting precision.

One comparative study evaluated the effectiveness of a deep feedforward neural network against an artificial neural network (ANN) for predicting stock index prices, demonstrating the potential of deep learning in this context^3^. Another approach involves using regression analysis through a multilayer perceptron (MLP), an operational ANN model capable of estimating stock market prices^4^. Among the best time series prediction models is the Adaptive Neuro-Fuzzy Inference System (ANFIS), which has been successfully applied in numerous forecasting tasks^5–7^.

Zheng et al. proposed a novel model called Random Long Short-Term Memory (RLSTM), which builds on the Long Short-Term Memory (LSTM) architecture while addressing the problem of overfitting in stock market index forecasting. By incorporating a sequence of random data with a uniform distribution, RLSTM significantly outperforms conventional models in terms of prediction accuracy^8^.

Another innovative approach by Chen et al. combined public mood and emotion data with predictive computing to enhance stock market trend forecasting. This model demonstrated superior accuracy compared to traditional machine learning techniques, highlighting the value of integrating sentiment analysis into financial predictions^9^.

Yeh et al. employed a multiple kernel support vector regression (MKSVR) method to estimate stock market prices, achieving improved predictive performance^10^. Similarly, Singh et al. introduced a hybrid model known as Wavelet-Based Soft Computing (WSC) to increase forecast accuracy, allowing investors to make more profitable decisions based on reliable stock market estimates^11^.

In a comparative study focused on classification efficiency, the Support Vector Machine (SVM) technique outperformed ANFIS in detecting finger vein patterns, demonstrating greater computational efficiency and classification power^12^.

Additionally, Liao et al. proposed a transformer model designed to improve the acquisition of positional information related to long-term emotions. By incorporating ProbAttention and rotating position encoding, the Long-Term Sentiment Change Enhanced Temporal Analysis (LEET) method was validated using the S&P 500 (SP500) and FTSE 100 indexes. Experimental results indicated that LEET outperformed most deep learning architectures commonly used for stock price prediction^13^.

These advancements underscore the ongoing evolution of AI-based financial forecasting, particularly the integration of hybrid and deep learning models to improve prediction accuracy and computational efficiency.

Recent studies have explored various optimization techniques for financial forecasting, including the use of dendrite neuron models and median dendritic artificial neural networks (Cansu et al.^32^; Bas et al.^33^).

Several researchers are looking into optimization techniques to improve the effectiveness of current machine learning algorithms. Lu in^14^ used PSO to optimize the SVR model’s parameters to make a hybrid stock index forecast model. Shen et al. suggested an optimized a radial basis function NN using the artificial fish swarm algorithm (AFSA) to estimate the Shanghai Security Exchange index^15^. Gülmez in^16^ provided a novel LSTM network model for stock market forecast that was optimized LSTM using the Artificial Rabbits Optimization algorithm (ARO). The best model among the models is the LSTM-ARO model, according to the results. Das et al. presented a method to modify the Crow Search Algorithm (CSA) and Extreme Learning Machine (ELM)^17^. Also, the authors used as PSO depended on Group oriented CSA (PGCSA) techniques. A thorough comparison with some other models in^18,19^ revealed that the modified method, PGCSA ELM is superior. Qiu et al. applied an attention based Long Short Term Memory NN and a wavelet transform in order to forecast of the stock price. The authors also implemented their method and compared it to other models and showed that the forecasting results from their offered model are more accurate^20^. Chen and Zhou applied the first technique a Genetic Algorithm (GA) to select the best features, used datasets from the China Construction Bank and the CSI 300 stock index. Moreover, the authors applied the model GA-LSTM, a combination of GA and LSTM, to facilitate efficient feature selection and modeling of intricate stock price dynamics^21^.

HHO is introduced by Heidari et al. in 2019, a population-based meta-heuristic method. Through the sharing of knowledge amongst Harris Hawks, HHO carries out the best hunting plan and successfully catches the prey^22^. Some of the shortcomings with the HHO approach include falls into a local optimum solution and sluggish convergence speeds. The population-based optimization technique is recognized to be modifiable in order to search for more precise solutions and avoid various local optima. To overcome these drawbacks and improve the intensification and diversification processes, the dynamical properties of chaos maps were extensively employed. In search procedures and speed parameters, the standard HHO is combined with the chaotic mapping mechanism to achieve this goal. HHO make use of the prey’s earlier unknown position^23^. Based on algorithmic behavior, the Chaotic Harris Hawk Optimization (ChHHO) is developed. Moreover, chaotic variables are used in place of the random values in. Once combined with chaotic variables, HHO’s exploration is enhanced through the application of the Levy distribution.

ANFIS used for many forecasting challenges and is among the best time series forecasting methods^5–7^. However, there are some shortcomings in the classical ANFIS model’s parameters design. Parameters design are a useful step that influence the performance of solution. Therefore, most of the optimization methods are employed to improve the parameters design process.

The novelty of the proposed ANFIS-ChHHO model lies in its unique integration of the Adaptive Neuro-Fuzzy Inference System (ANFIS) with the Chaotic Harris Hawks Optimization (ChHHO) algorithm. Unlike conventional models, the proposed approach enhances prediction accuracy by addressing the local optima problem often encountered in stock price forecasting. The chaotic mapping mechanism incorporated within the HHO framework significantly improves the exploration and exploitation balance, thus optimizing the ANFIS parameters more effectively. This hybrid model outperforms existing techniques by yielding lower error rates and more reliable predictions, particularly in volatile financial environments. Such advancements demonstrate a significant contribution to the field of stock market prediction, addressing critical challenges inherent in time series forecasting.

In order to get over classical ANFIS limitations, this paper uses ChHHO method to optimize ANFIS parameters in order to get over classical ANFIS limitations. We proposes an optimized ANFIS model that includes the modified HHO meta-heuristic optimization algorithm. The enhanced HHO algorithm is proposed to improve the effectiveness of ANFIS in stock price forecasting. Using stock marketing in Egypt datasets, the ANFIS-ChHHO model’s effectiveness is evaluated and compared with other modified ANFIS models that use some optimization strategies as well as standard ANFIS models. The results indicate that the ANFIS-ChHHO model performs better in stock price prediction than both the other used models and the classical ANFIS model.

The following summarizes this paper’s main contributions:

(i) It includes the introduction of the ANFIS-ChHHO model, a stock price time series prediction model.
(ii) The developed HHO algorithm to optimize ANFIS parameters in order to get over ANFIS’s limitations.
(iii) It evaluates and compares of ANFIS-ChHHO with other models using three datasets. (iv) It optimizes ANFIS parameters using the proposed ANFIS-ChHHO approach in order to improve forecasting precision for the stock prices.

The rest of this work is structured as the following: in Section "Preliminaries", the classical ANFIS and HHO algorithm are discussed. The proposed model ANFIS-ChHHO for predicting stock price is shown in Section "The improved method (Quadratic Chaotic HHO)". The experiments and their results are displayed in Section "Experiments". Lastly, the conclusions and suggested future work are presented in Section "Results".

## Preliminaries

### Adaptive neuro-fuzzy inference system (Anfis)

**Fig. 1:**
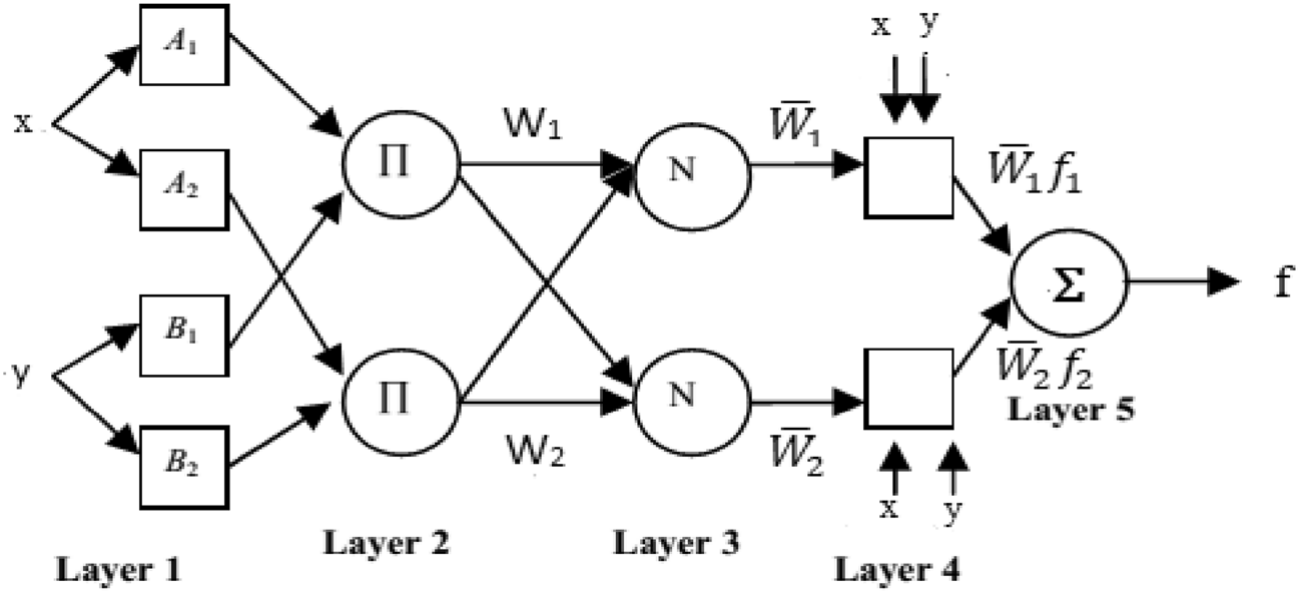
Classical ANFIS architecture.

The ANFIS model was presented by Jang in 1993^24^ as a combination of two approaches artificial neural networks and fuzzy inference system, it can manage non-linear and complex problems within a structure. Artificial neural networks bring the ability to learn and adapt from data, while fuzzy logic system allows the representation of human-like reasoning and decision-making. The Takagi–Sugeno model is considered the most often utilized fuzzy system in ANFIS model because it is more clear and requires less computing effort than other models. This model operates using the condition IF–THEN logic. ANFIS usually includes five layers as displayed in Fig. 1. Figure 1 describes the fundamental architecture of the ANFIS model which includes five layers. That, the inputs layer1 are x and y, and O_1i_ specifies the node i’s output. The classical ANFIS model can be expressed by Eqs. (1) and (2) as the following:

1 $${O}_{1i}={\mu }_{{A}_{i}} \left(x\right), i= \text{1,2} {O}_{1i}={\mu }_{{B}_{i}}\left(y\right), i=\text{1,2}$$

2 $$\mu \left(x\right)={e}^{{-\left(\frac{x-{\rho }_{i}}{{\alpha }_{i}}\right)}^{2}}$$

wherever, Every node produces the following output: O_1i_ that represents the membership (Ms) grade for inputs x and y. That, μ (x) is the Generalized Gaussian Ms function, while A_i_ and B_i_ are the Ms values of μ(x) . Moreover and α_i_ and ρ_i_ display the parameters of the premise. Also, the second layer can be displayed from Eq. (3) as the following:

3 $${O}_{2i}={\mu }_{{A}_{i}} \left(x\right)\times {\mu }_{{B}_{i}}\left(y\right) i=\text{1,2}$$

Furthermore, we define the third layer output as follows from Eq. (4), and this layer includes fixed nodes that compute from the previous layer.

4 $${O}_{3i}= \overline{{w}_{i}}= \frac{{\omega }_{i}}{{{\omega }_{1}+\omega }_{2}} i=\text{1,2}$$

Also, the layer 4 nodes are adaptive and apply the following rules: known as the consequent parameters, these parameters are q_i_, p_i_ and r_i._ of node i as in Eq. (5):

5 $${O}_{4i}= \overline{{w}_{i}}{f}_{i}= \overline{{w}_{i}}( {p}_{i}x+{q}_{i}y+{r}_{i})$$

Lastly, the fifth layer output is calculated as follows from Eq. (6).

6 $${O}_{5i}=\sum_{i}\overline{{w}_{i}}{f}_{i}$$

### Harris Hawk Optimization Algorithm (HHO)

HHO is a meta-heuristic algorithm that is a population-based algorithm presented by Heidari et al. in 2019^22^. HHO executes the optimal hunting strategy through the information exchange between Harris Hawks and captures the prey. This algorithm consists of three phases are exploration, conversion between exploration and exploitation, and exploitation. The detailed of these stages are as follows:

#### The first step (Exploration)

During this step, Hawks focus in a search area to hunt preys within the range of the population . Hawks use two methods for finding prey. The first strategy involves perching near other family members when q < 0.5. The second strategy involves randomly choosing a perch on a high pole or tree within their familiar area when q ≥ 0.5. The hawk’s position is updated during the exploration phase using Eq. (7).

7 $${z}_{m}\left(t+1\right)=\left\{\begin{array}{c}{\text{z}}_{r}\left({\text{t}}\right)-{\text{r}}_{1}\left|{\text{z}}_{r}\left({\text{t}}\right)-{2\text{r}}_{2} z\left(t\right)\right|, q\ge 0.5\\ {(\text{z}}_{prey}\left({\text{t}}\right)-{\text{z}}_{m}\left({\text{t}}\right))-{\text{r}}_{3}(L+ {\text{r}}_{4}\left( U-L\right)), q<0.5\end{array}\right.$$

8 $${z}_{m}\left(t\right)=\frac{1}{M}{\sum }_{k=1}^{M}{z}_{k}(t))$$

where z(t + 1) indicates the position of the hawks in the next iteration. z_prey_(t) denotes the place of the prey. z(t) denotes the location of the hawks in the current iteration t, r_1_ – r_4_ and q are randomly between 0 and 1. The upper and lower boundaries of the population are represented by U and L, in that order. z_r_ (t) indicates a hawk selected at random from the present population. The average of the individual positions in the present population, as given in Eq. (8), is presented as z_avg_ (t), N is the number of hawks.

#### The second step: transition phase between exploration and exploitation

In this step, The HHO method uses the prey’s escaping energy to switch between exploration and exploitation stages. This step used two search approaches through the prey’s escape energy EE′, when EE′ ≥ 1 the method selects exploring the search space for new solutions and when EE′ < 1 it switches to find the best solutions found during exploration, the Eq. (9) is as:

9 $$EE^{\prime}=2{E}_{0}\left(1-\frac{t}{T}\right)$$

10 $$E_{0} = 2*r_{5} - 1$$

where, EE′ denotes the escaping energy of the prey at a certain iteration. E_0_ as in Eq. (10) shows the initial value of energy of the prey. The t and T are the current iteration number and the maximum number of iterations respectively.

#### The third step (Exploitation)

Through, this stage involves searching and monitoring prey behavior, waiting for the optimal time to attack. The exploitation phase results the actual attack, where the hawk utilizes four various approaches to capture its prey. The process switches from exploration to exploitation when a specific energy level (EE′) drops below a threshold (|EE′|< 1). The hawk’s strategy depends on the prey’s ability to escape, signified by a random number (r) between 0 and 1.

##### First strategy

Soft Siege, the victim attempts to get away from the chase by skipping, if (|EE′|≥ 0.5 & r ≥ 0.5), the HH can evenly bounded it, fatiguing the prey and then held it. This procedure is demonstrated as follows in Eqs. (11) and (12):

11 $$z\left( {t + 1} \right) = z\left( t \right) - EE^{\prime } \left| {Jz_{prey} \left( t \right) - z\left( t \right)} \right|$$

where

12 $$\Delta z\left({\text{t}}\right)={\text{z}}_{prey}\left({\text{t}}\right)- z\left(t\right)\text{ and }{\text{J}}=2(1-{r}_{5})$$

That, $\Delta z\left({\text{t}}\right)$ shows the difference between the prey’s position and its current position and J denotes the random jump power of the prey.

##### Second strategy

Hard Siege, in this scenario the prey’s energy is low and the hawk can attack the prey strongly, if (r ≥ 0.5 & |EE′|< 0.5). This step is showed in Eq. (13) is as:

13 $$z\left(t+1\right)={\text{z}}_{prey}\left(t\right)-EE{\prime} \left|\Delta z\left({\text{t}}\right)\right|$$

##### Third strategy

Progressively rapid dives during a soft besiege, in this case, the victim has mandatory energy to get away, If (r < 0.5 & |EE′|≥ 0.5), but the hawk uses the skilled approach is called Levy flight (Lf) to trace irregular motions of the prey. The hawk dives fast and unpredictable to right its position and show the best changes. This approach is displayed as the following:

Where, *u*, *v* are random values belongs to (0,1), β = 1.5, X and Y are computed by Eqs. (14) and (15).

##### Third strategy

Progressively rapid dives during a soft besiege, in this case, the victim has mandatory energy to get away, If (r < 0.5 & |EE′|≥ 0.5), but the hawk uses the skilled approach is called Levy flight (Lf) to trace irregular motions of the prey. The hawk dives fast and unpredictable to right its position and show the best changes. This approach is displayed as the following:

14 $$X = z_{prey} \left( t \right) - EE^{\prime } \left| { J z_{prey} \left( t \right) - z\left( t \right)} \right|$$

15 $$Y = X + S. LF\left( D \right)$$

16 $$LF\left( z \right) = 0.01 \frac{u \times \sigma }{{\left| v \right|^{{\frac{1}{\beta }}} }}, \sigma = \left( {\frac{{\Gamma \left( {1 + \beta } \right) \times \sin \left( {\frac{\pi \beta }{2}} \right)}}{{\Gamma \left( {\frac{1 + \beta }{2}} \right) \times \beta \times 2^{{\left( {\frac{\beta - 1}{2}} \right)}} }}} \right)^{{\frac{1}{\beta }}}$$

where, *u*, *v* are random values belongs to (0,1), β = 1.5, X and Y are computed by Eqs. (14) and (15).

17 $$z(t+1)=\left\{\begin{array}{c}X if F\left(X\right) < F(z\left(\text{t}\right))\\ Y if F\left(Y\right) < F(z\left(\text{t}\right))\end{array}\right.$$

wherever Lf( ) is the function of levy flight, Rvec is a random vector, and Dim is the dimension of the stated problem. The location of the Hawks is updated from Eq. (17).

2.2.3.4 The fourth part: hard besiege with rapid diving, if (r < 0.5 & |EE′|< 0), the prey is very fatigued to flee and the hawk surrounds it. The best time to the hawk is decide by the average of the locations of the prey, which is given in Eqs. (20).

18 $$X^{\prime } = z_{prey} \left( t \right) - EE^{\prime } \left| { J \times z_{prey} \left( t \right) - z_{m} \left( t \right)} \right|$$

19 $$Y^{\prime } = X^{\prime } + S \times LF\left( D \right)$$

20 $$z(t+1)=\left\{\begin{array}{l}X^{\prime} if F\left(X^{\prime}\right) < F(z\left(\text{t}\right))\\ Y^{\prime} if F\left(Y^{\prime}\right) < F(z\left(\text{t}\right))\end{array}\right.$$

where X and Y${\prime}$ are computed by Eqs. (18) and (19), z_avg_(t) is found in Eq. (8).

In the following section, we show the improved technique.

## The improved method (Quadratic Chaotic HHO)

In this section, we presented an improved HHO algorithm, which is based on chaos. In deterministic nonlinear systems, chaos is a restricted dynamic behavior characterized by nonlinear systems, unpredictable behavior and sensitivity. The use of chaotic systems in meta-heuristic algorithms for the initial population has increased recently. Some of the shortcomings with the HHO approach include falls into a local optimum solution and slow convergence speeds. Our objective, we improve the population initialization and the exploration capabilities of the method, the enhanced algorithm can accomplish a superior balance between the exploration phase and exploitation to get the best solution. HHO use the prey’s position, which is unknown earlier^23^. Based on algorithmic behavior, we have created the Chaotic HHO (ChHHO). Additionally, chaotic variables are used in place of the random values in ChHHO. HHO makes use of the Levy distribution, which improves the HHO’s exploration when combined with chaotic variables. Enhancing the initial population of HHO is the goal of ChHHO in order to improve the average population which lead to an efficient search process and better convergence.

From the types of used chaotic maps as a logistic, a tent, a Chebyshev, a Gauss, a Circle, and a Sine chaotic maps^25,26^. Among these types, it was found that logistic chaotic map is better in convergence and ergodicity^25^. Yuan et al., used Singer, Gauss, and tent chaotic maps to enhance the technique’s performance^25^. In this work, we used the quadratic chaotic map to enhance the population initialization and the exploration capabilities of our approach as showed in Eq. (21).

21 $$x_{i + 1} = a - x_{i}^{2} l,\;a = 1.4$$

where x_i_ is a control parameter, and the value range is (0,1), a is constant equal 1.4 as in^27^.

The uniqueness of the proposed ANFIS-ChHHO algorithm lies in its ability to dynamically adjust the search space by integrating chaotic variables. This not only prevents premature convergence but also improves prediction stability, making it more effective for volatile financial data.

Algorithm 1 demonstrates the improved Harris Hawk Optimization method.

**Algorithm 1:**
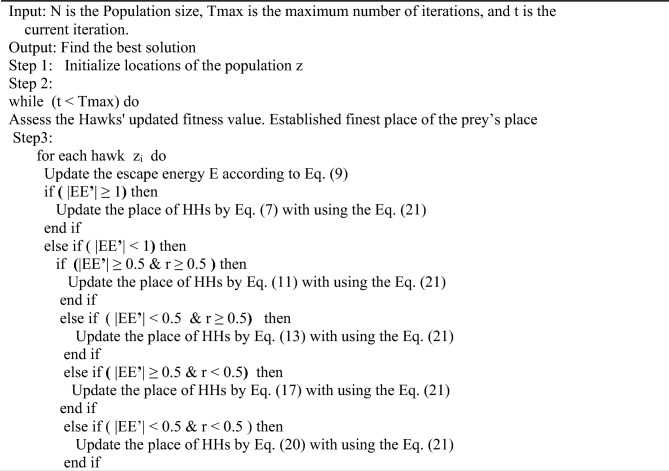
The framework of the proposed model ANFIS – ChHHO.

### The workflow of the ANFIS-ChHHO model

**Fig. 2:**
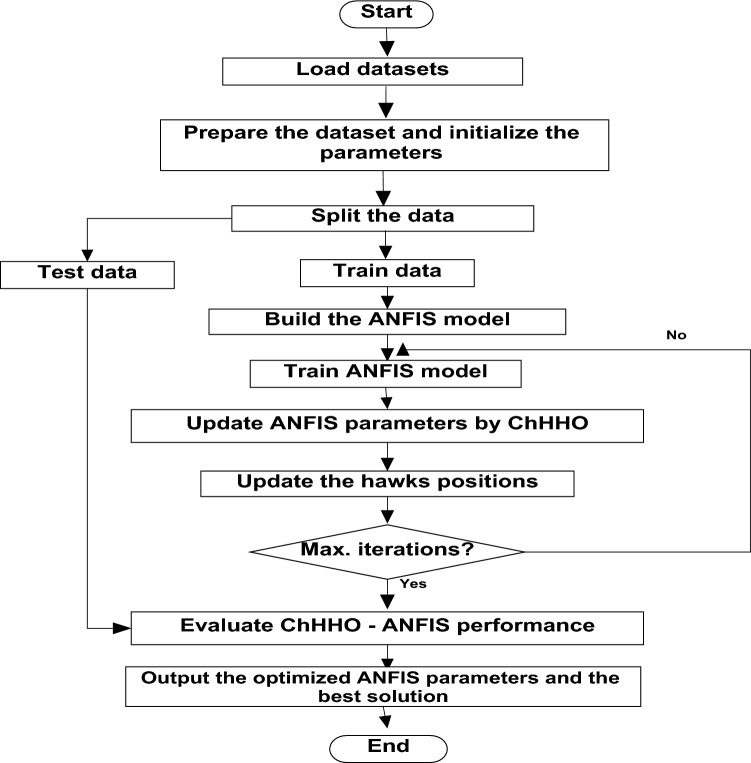
The framework of the proposed model ANFIS – ChHHO.

This section presents the proposed ANFIS-ChHHO model for stock market prediction. The parameters of ANFIS are optimized with the ChHHO methodology, which enhances the efficacy of the HHO method. The ANFIS model is optimized using the improved HHO (ChHHO) approach for stock market forecasting. The steps are illustrated in Fig. 2. In the ANFIS-ChHHO model, the initial phase involves partitioning the stock market data into two subsets: the training set, including 70% of the whole data, and the testing set, which contains 30% of the overall data In the following step, the improved algorithm is used to search for the optimal parameters of the ANFIS model. Lastly, the ANFIS model with the optimal parameters is used to predict of the stock.

The main steps of framework of the proposed model ANFIS-ChHHO:

Step 1: Prepare the datasets and initialize the parameters.

Step 2: Divide the data into training and testing.

Step 3: Design the ANFIS network, train the ANFIS network and calculate the expected output of the training data as shown in the Eq. (22) .

22 $$\text{MSE}= \frac{1}{\text{N}} \sum_{\text{i}=1}^{\text{N}}(\text{X}_{\rm i}-{\text{PX}_{\rm i})}^{2}$$

That, X_i_ is actual value, PX_i_ is expected value and N is number of samples.

Step 4: Update ANFIS model by using the improved HHO.

Step 5: Evaluate the current solution, checked the terminal condition and repeated the steps until the end of the condition.

Step 6: The training result is tested with the test data.

Step 7: The best ANFIS model is used for prediction of the stock.

## Experiments

To clearly highlight the effectiveness of the proposed model, we present a comparative analysis using newly introduced performance metrics such as RMSRE, MSRE, MARE, and RMSPE. Additionally, structured subheadings were added to delineate the results for each dataset (EGX-30, EGX-70, EGX-100). Each subsection now includes detailed comparative tables and graphical illustrations to visualize the performance differences. The inclusion of real-world stock prediction scenarios further demonstrates the practical implications of the proposed model, showing its potential in financial decision-making processes.

### Datasets

In this work, we apply on the Egyptian Stock Exchange (EGX ) on the historical data for three stock indices: the EGX-30 Index (EGX30), EGX-70 Index (EGX70), and EGX-100 Index (EGX100). These datasets are from January 3, 2010, to June 27, 2019. These data are collected from (https://www.investing.com). The basic transaction datasets contain of six variables. The feature of open price present the value at which a security is implemented per share when the market opens while the feature of close price is the value at which a security is managed per share when the market close. A stock’s daily high and low are its highest and lowest values, in that order. The quantity of a operation in a given amount of time is mentioned as its volume. The datasets include six features: date, open, high, low and close prices and the volume as presented in Table 1. This work employs four features: open, high, and low prices as input variables of the proposed model ANFIS-ChHHO for evaluating the close price of the following day.

**Table 1:** Partial stock data samples for EGX-30.

Date	Open	High	Low	Close	Volume
1/3/2010	6,208.77	6,273.73	6,208.77	6,272.00	41,352,416
1/4/2010	6,272.00	6,333.31	6,272.00	6,323.90	70,571,761
1/5/2010	6,323.90	6,430.37	6,323.90	6,420.49	57,499,851
…	…	…	…	…	…
6/26/2019	13,831.23	14,006.99	13,828.36	14,006.99	250,715,304
6/27/2019	14,006.99	14,150.08	14,006.99	14,100.74	251,880,306

### Performance metrics

Four evaluation metrics are employed in this study as Mean Absolute Error (MAE), Root Mean Square Error (RMSE), and Standard deviation (SD)^28–31^. The smallest values of these metrics demonstrate better predicted data. Also, Theil’s U is used to determine the predicting accuracy. The minimum value of Theil’s U demonstrates more accuracy of prediction^17^. These metrics are computed by the following Eqs. (23)–(26):

23 $$MAE = \frac{1}{N}\mathop \sum \limits_{i = 1}^{N} \left| {Y_{i} - PY_{i} } \right|$$

24 $$RMSE=\sqrt{\frac{1}{N} \sum_{i=1}^{N}{({Y}_{i} - P{Y}_{i})}^{2}}$$

25 $$SD=\frac{1}{N} \sum_{i=1}^{N}{({Y}_{i} - {\overline{Y} }_{i})}^{2}$$

26 $${\text{Theil}}'{\mathrm{s}}\;U = \frac{{\sqrt {\frac{1}{N} \mathop \sum \nolimits_{i = 1}^{N} \left( {Y_{i} - PY_{i} } \right)^{2} } }}{{\sqrt {\frac{1}{N} \mathop \sum \nolimits_{i = 1}^{N} \left( {Y_{i} } \right)^{2} } + \sqrt {\frac{1}{N} \mathop \sum \nolimits_{i = 1}^{N} \left( { PY_{i} } \right)^{2} } }}$$

The new metrics in Eqs. (27)–(29):

27 $$MSRE=\frac{1}{N} \sum_{i=1}^{N}{\{{Y}_{i} - P{Y}_{i})/{Y}_{i}\}}^{2}$$

28 $$RMSRE=\sqrt{\frac{1}{N} \sum_{i=1}^{N}{\{{(Y}_{i} - P{Y}_{i})/{Y}_{i}\}}^{2}}$$

29 $$RMSRE=\sqrt{\frac{1}{N} \sum_{i=1}^{N}{\{{Y}_{i} - P{Y}_{i})/{Y}_{i}\}}^{2}*100}$$

30 $$MARE = \frac{1}{N}\mathop \sum \limits_{i = 1}^{N} \left| {(Y_{i} - PY_{i} )/Y_{i} } \right|$$

The metrics are MARE, MSRE, RMSRE, RMSPE are refer to Mean Absolute Relative Error, Mean Squared Relative Error, Root Mean Squared Relative Error and the last is Root Mean Squared Percentage Error and these metrics the minimum is better.

Where ${\text{Y}}_{\text{i}},$ ${\text{PY}}_{\text{i}}$ are the values of target and predicted respectively. $\overline{{\text{Y} }_{\text{i}}}$ is the mean value of target. $N$ is number of samples.

### Comparison to other predicting approaches

In this study, the ChHHO-ANFIS compared to some meta-heuristics algorithms as HHO algorithm, particle swarm optimization algorithm (PSO), grey wolf optimization algorithm (GWO), and the classical ANFIS models. The dataset is divided into 70% for training and 30% for testing in all work. Table 2 presents the settings for all techniques, which were executed separately several times and the CPU is Intel (R) Core (TM) i7-6600U CPU @ 2.60GHz 2.80 GHz, MATLAB 2016a is used for all operations.

**Table 2:** Parameter setting.

Algorithm	Parameter value	Value
Common parameters	Population size	50
	Max iterations	1000
	Lower bound	− 25
	Upper bound	25
HHO	Beta	1.5
PSO	c1	1
	c2	2
	W	1
	W_damp_	0.99
GWO	A	[2:0]
ChHHO	Beta	1.5
	Initial value of chaotic map	0.6

## Results

### Results of EGX-30

**Fig. 3:**
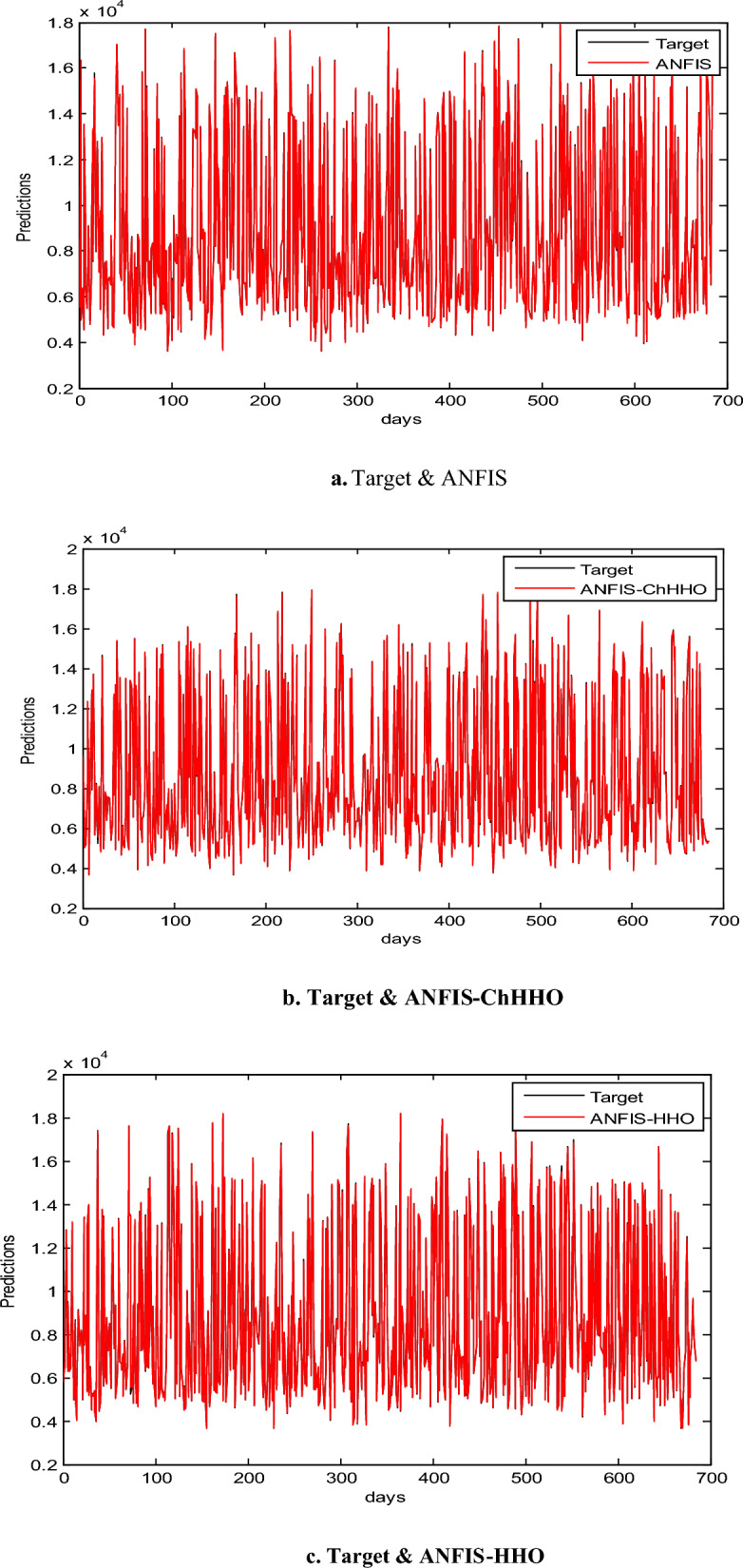

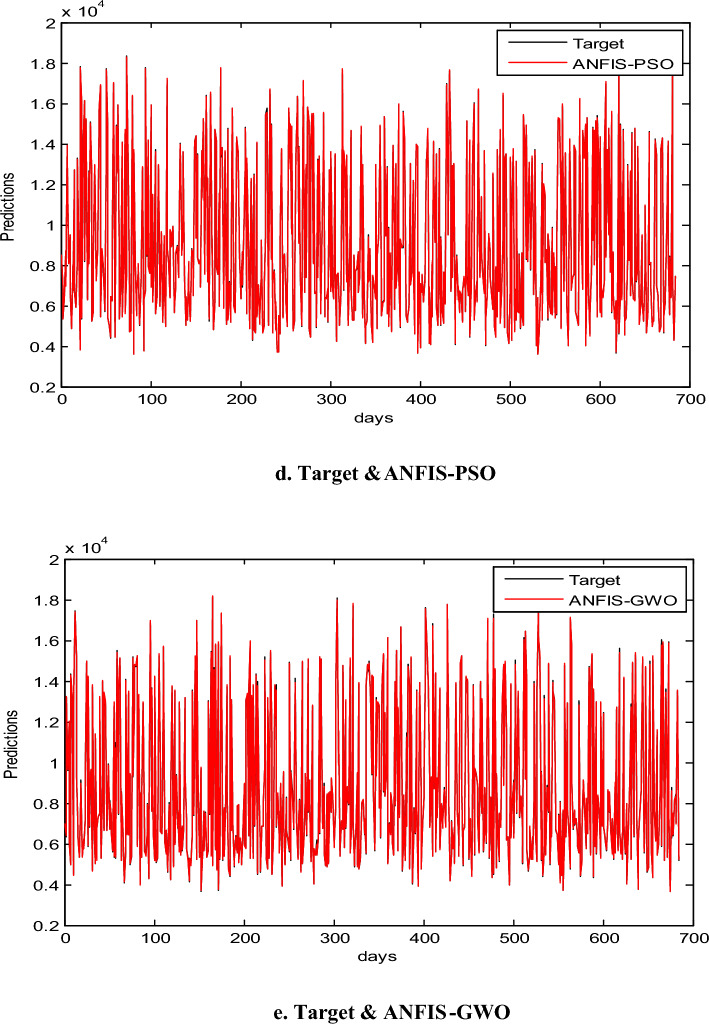
Prediction results of EGX-30 dataset.

**Fig. 4:**
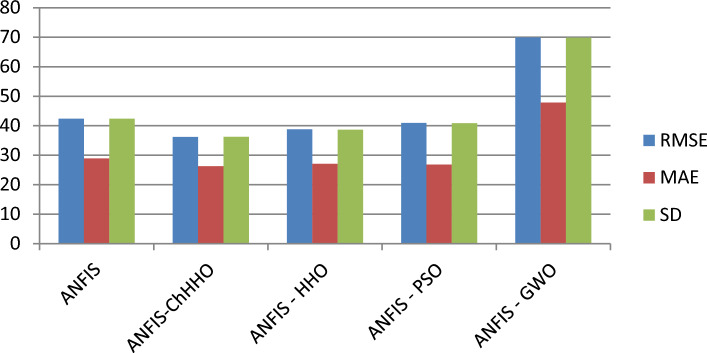
The results of metrics EGX-30 for testing the five models.

In this section, we use the EGX-30 a first stock marketing datasets to evaluate the proposed ANFIS-ChHHO technique. We considered several well-known optimization algorithms to be compared the proposed method, including the classical ANFIS model as the standard HHO algorithm, PSO algorithm and GWO algorithm. The findings of all models record in Table 3 about, MAE, RMSE, SD and Theil’s U. We can see that the ANFIS-ChHHO accomplished the best MAE value, then came ANFIS-HHO, ANFIS, ANFIS-PSO and ANFIS-GWO model in that order. Also, the ANFIS-ChHHO also got the best RMSE value, where ANFIS-HHO obtained the second rank. Other models came in the following order, ANFIS, ANFIS-PSO and ANFIS-GWO**.** Further, the ANFIS-ChHHO attained the best Theil’s U value of 0.0019, and ANFIS model got the second rank, and two models obtained the third rank, ANFIS- HHO and ANFIS-PSO, and ANFIS-GWO model attained the fourth rank. More, the ANFIS- ChHHO come in the first rank in the measured of SD, after that ANFIS-HHO, ANFIS, PSO-ANFIS, and GWO-ANFIS respectively. Also, Figs. 3a–e and 4 demonstrate the results of prediction of the next closing price day of the ANFIS-ChHHO compared to other models. It is clear that our method is better than other models with the nearest values to the target data. Figure 4 illustrates the results of the RMSE, MAE, and SD metrics for testing the five models (AMF5, AMF5-CHH4O, AMF5-HHO, AMF5-PSO, and AMF5-GWO) on the EGX-30 dataset.

To further validate the robustness of the proposed model, we calculated additional performance metrics: RMSRE, MSRE, MARE, and RMSPE. These metrics provide a more comprehensive evaluation of prediction accuracy. As depicted in Tables 3, 4, the ANFIS-ChHHO model consistently outperformed conventional models across all metrics, indicating its superior generalization ability.

**Table 3:** Prediction performance of different models (ANFIS, ANFIS-ChHHO, ANFIS-HHO, ANFIS-PSO, ANFIS-GWO) on the EGX-30 index testing set: RMSE, MAE, SD, and Theil’s U.

	RMSE	MAE	SD	Theil’s U
ANFIS	40. 687	27.8522	40.7167	0.0021
ANFIS-ChHHO	38.2695	27.2965	38.2711	0.0019
ANFIS-HHO	39.1942	27.6107	39.1073	0.0022
ANFIS-PSO	42.639	28.3004	42.6701	0.0022
ANFIS-GWO	64.9128	47.495	64.9603	0.0033

Bold indicates the best results.

OR.

If used these metrics only.

**Table 4:** Prediction performance of different models (ANFIS, ANFIS-ChHHO, ANFIS-HHO, ANFIS-PSO, ANFIS-GWO) on the EGX-30 index testing set: MARE, MSRE, RMSRE, and RMSPE.

	MARE	MSRE	RMSRE	RMSPE
ANFIS	0.003234	2.0298e5	0.0045054	0.45054
ANFIS-ChHHO	0.002792	1.9824e-5	0.0044524	0.44524
ANFIS-HHO	0.003692	5.9799e-5	0.007733	0.7733
ANFIS-PSO	0.003399	2.5649e-5	0.0050645	0.50645
ANFIS-GWO	0.003371	2.4951e-5	0.0049951	0.49951

Bold indicates the best results.

OR.

Additionally metrics as.

The predictive performance of various ANFIS-based models (ANFIS, ANFIS-ChHHO, ANFIS-HHO, ANFIS-PSO, and ANFIS-GWO) on the EGX-30 index testing set is systematically evaluated across multiple error metrics. Table 3 compares the models using traditional accuracy measures (RMSE, MAE, SD, and Theil’s U), revealing ANFIS-ChHHO’s superior performance with the lowest RMSE (38.2695) and Theil’s U (0.0019). Table 4 extends this analysis to relative error metrics (MARE, MSRE, RMSRE, and RMSPE), where ANFIS-ChHHO again demonstrates robustness, particularly in MARE (0.002792). Finally, Table 5 consolidates all eight metrics, providing a comprehensive view of model performance; notably, ANFIS-ChHHO maintains consistently competitive results across all measures, while ANFIS-GWO exhibits the highest errors (e.g., RMSE: 64.9128, Theil’s U: 0.0033), highlighting the impact of optimization techniques on prediction accuracy.

**Table 5:** Performance metrics of ANFIS-based models (ANFIS, ANFIS-ChHHO, ANFIS-HHO, ANFIS-PSO, ANFIS-GWO) on EGX-30 index testing set: RMSE, MAE, SD, Theil’s U, MARE, MSRE, RMSRE, and RMSPE values.

	ANFIS	ANFIS-ChHHO	ANFIS-HHO	ANFIS-PSO	ANFIS-GWO
RMSE	40. 687	38.2695	39.1942	42.639	64.9128
MAE	27.8522	27.2965	27.6107	28.3004	47.495
SD	40.7167	38.2711	39.1073	42.6701	64.9603
Theil’s U	0.0021	0.0019	0.0022	0.0022	0.0033
MARE	0.003234	0.002792	0.003692	0.003399	0.003371
MSRE	2.0298e5	1.9824e-5	5.9799e-5	2.5649e-5	2.4951e-5
RMSRE	0.0045054	0.0044524	0.007733	0.0050645	0.0049951
RMSPE	0.45054	0.44524	0.7733	0.50645	0.49951

Bold indicates the best results.

### Results of EGX-70

**Fig. 5:**
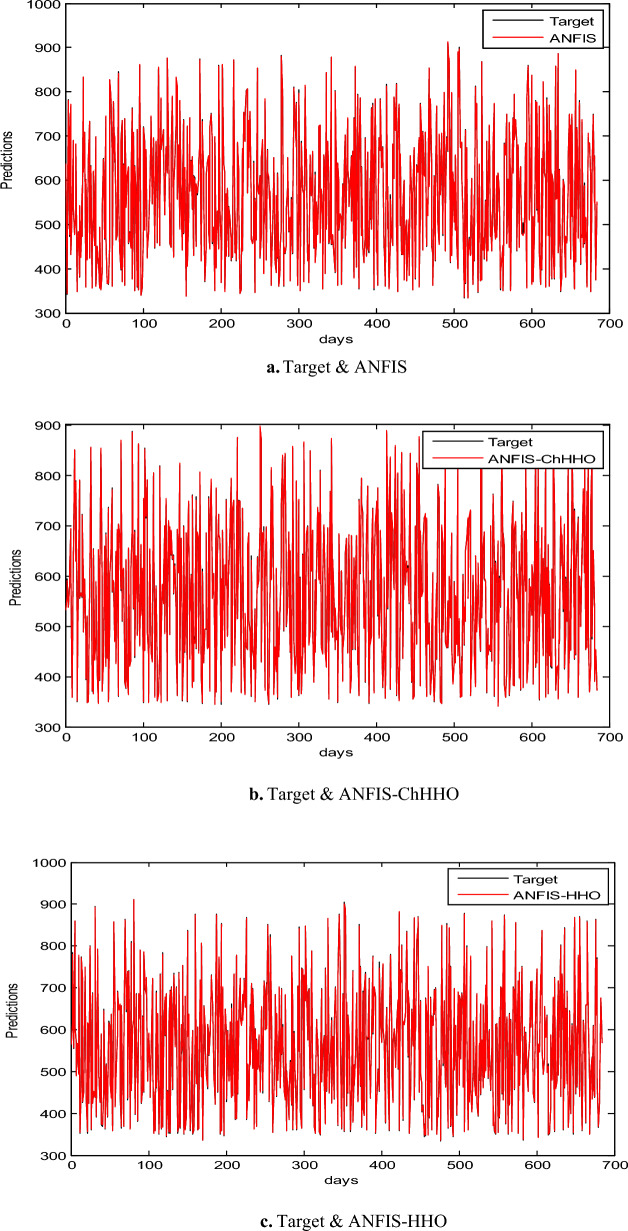

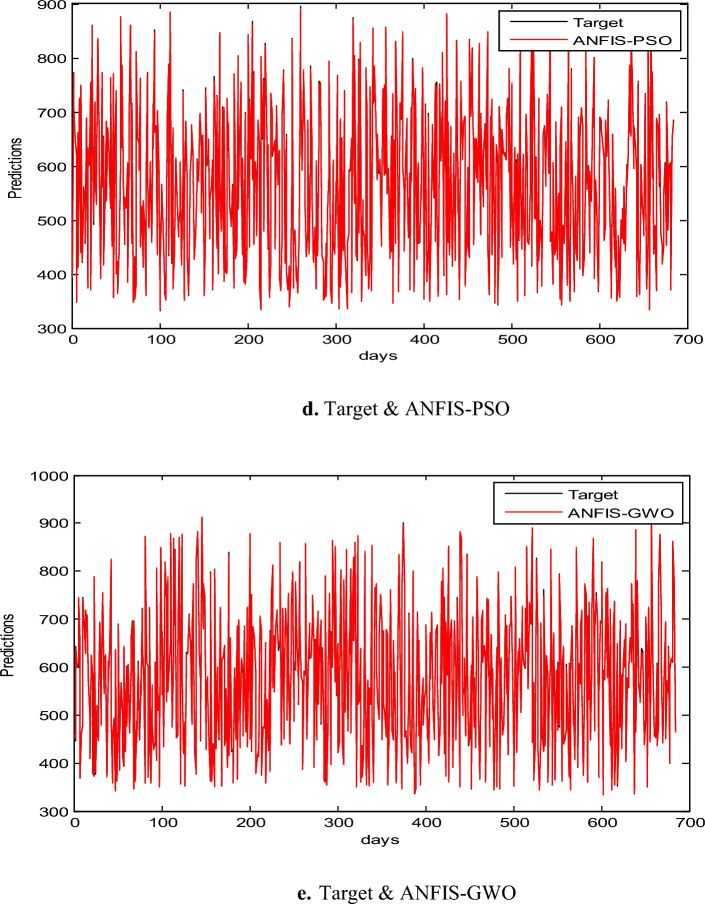
Prediction results of EGX-70 dataset.

**Fig. 6:**
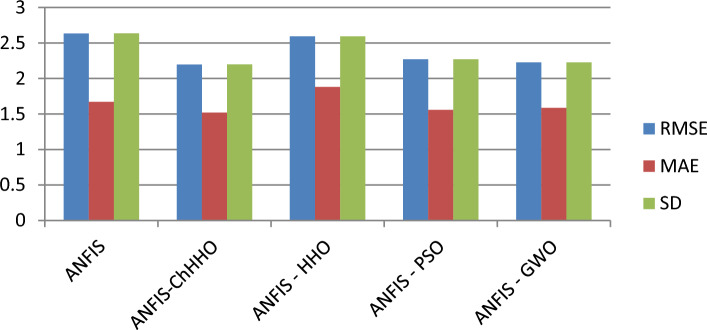
The results of metrics EGX-70 for testing the five models.

In this part, we assess the proposed ANFIS-ChHHO method using the EGX-70 dataset. The findings of all models list in Table 4 about, MAE, RMSE, SD and Theil’s U. it is clear that the proposed approach got the best RMSE value, then ANFIS-HHO, ANFIS-PSO, ANFIS and ANFIS-GWO model, in that order. Also, ANFIS-ChHHO got the best MAE value, where ANFIS-PSO achieved the second rank. Other models as the following, ANFIS-HHO, ANFIS model and ANFIS-GWO. Further, the ChHHO-ANFIS attained the best Theil’s U value of 0.0019, and two models obtained the second rank ANFIS-HHO and ANFIS-PSO, ANFIS model got the third rank, and ANFIS-GWO model obtained the fourth rank. Also, the ANFIS-ChHHO came in the first rank in measured of SD, followed by ANFIS-HHO, ANFIS-PSO, ANFIS and the ANFIS-GWO model. Furthermore, Figs. 5a–e and 6 display the results of the all methods and the ANFIS-ChHHO of prediction of the next closing price day of the proposed method to compare the other models. As saw that, the proposed method achieved the closest values to the real data.

The predictive performance of ANFIS and its hybrid variants on the EGX-70 index is comprehensively evaluated across multiple metrics (Tables 6, 7, 8). Table 6 demonstrates ANFIS-ChHHO’s superiority in absolute error metrics (RMSE: 2.2414, MAE: 1.5336) and stability (Theil’s U: 0.0019), outperforming standard ANFIS by 13–15%. Table 7 further confirms this advantage in relative error measures, with ANFIS-ChHHO achieving the lowest MARE (0.002815) and RMSPE (0.40613), reflecting 9–33% improvements over other hybrids. The consolidated results in Table 8 reveal ANFIS-ChHHO’s consistent dominance across all eight metrics, particularly in MSRE (1.6824 × 10^−5^) and RMSRE (0.0040613), while ANFIS-PSO and ANFIS-GWO exhibit higher deviations. Together, these tables underscore the robustness of the ChHHO-optimized model for EGX-70 index forecasting.

**Table 6:** Performance comparison of different ANFIS-based hybrid models (ANFIS-ChHHO, ANFIS-HHO, ANFIS-PSO, ANFIS-GWO) and standalone ANFIS in predicting the EGX-70 index for the testing set, evaluated using RMSE, MAE, SD, and Theil’s U statistics.

	RMSE	MAE	SD	Theil’s U
ANFIS	2.5815	1.6791	2.5812	0.0022
ANFIS-ChHHO	2.2414	1.5336	2.2428	0.0019
ANFIS – HHO	2.3608	1.6421	2.3556	0.0020
ANFIS – PSO	2.384	1.6089	2.3821	0.0020
ANFIS – GWO	2.7444	1.7958	2.7464	0.0023

Bold indicates the best results.

OR.

If used these metrics only.

**Table 7:** Comparative performance of ANFIS and its hybrid variants (ANFIS-ChHHO, ANFIS-HHO, ANFIS-PSO, ANFIS-GWO) in predicting the EGX-70 index for the testing set, assessed using MARE, MSRE, RMSRE, and RMSPE metrics.

	MARE	MSRE	RMSRE	RMSPE
ANFIS	0.003104	1.8397e-5	0.0042892	0.42892
ANFIS-ChHHO	0.002815	1.6824e-5	0.0040613	0.40613
ANFIS-HHO	0.003063	2.0024e-5	0.0044748	0.44748
ANFIS-PSO	0.003139	2.0323e-5	0.0045081	0.45081
ANFIS-GWO	0.002948	1.9977e-5	0.0044696	0.44696

Bold indicates the best results.

If used these metrics additionally.

**Table 8:** Comprehensive performance evaluation of ANFIS and its hybrid variants (ANFIS-ChHHO, ANFIS-HHO, ANFIS-PSO, ANFIS-GWO) in forecasting the EGX-70 index across multiple error metrics (RMSE, MAE, SD, Theil’s U, MARE, MSRE, RMSRE, RMSPE) for the testing set.

	ANFIS	ANFIS-ChHHO	ANFIS-HHO	ANFIS-PSO	ANFIS-GWO
RMSE	2.5815	2.2414	2.3608	2.384	2.7444
MAE	1.6791	1.5336	1.6421	1.6089	1.7958
SD	2.5812	2.2428	2.3556	2.3821	2.7464
Theil’s U	0.0022	0.0019	0.0020	0.0020	0.0023
MARE	0.003104	0.00281	0.003063	0.003139	0.002948
MSRE	1.8397e-5	1.6824e-5	2.0024e-5	2.0323e-5	1.9977e-5
RMSRE	0.0042892	0.0040613	0.0044748	0.0045081	0.0044696
RMSPE	0.42892	0.40613	0.44748	0.45081	0.44696

Bold indicates the best results.

Figure 6 displays the results of metrics EGX-70 for testing the five models, while Fig. 5a–e are expressed the prediction results of EGX-70 dataset.

### Results of EGX-100

**Fig. 7:**
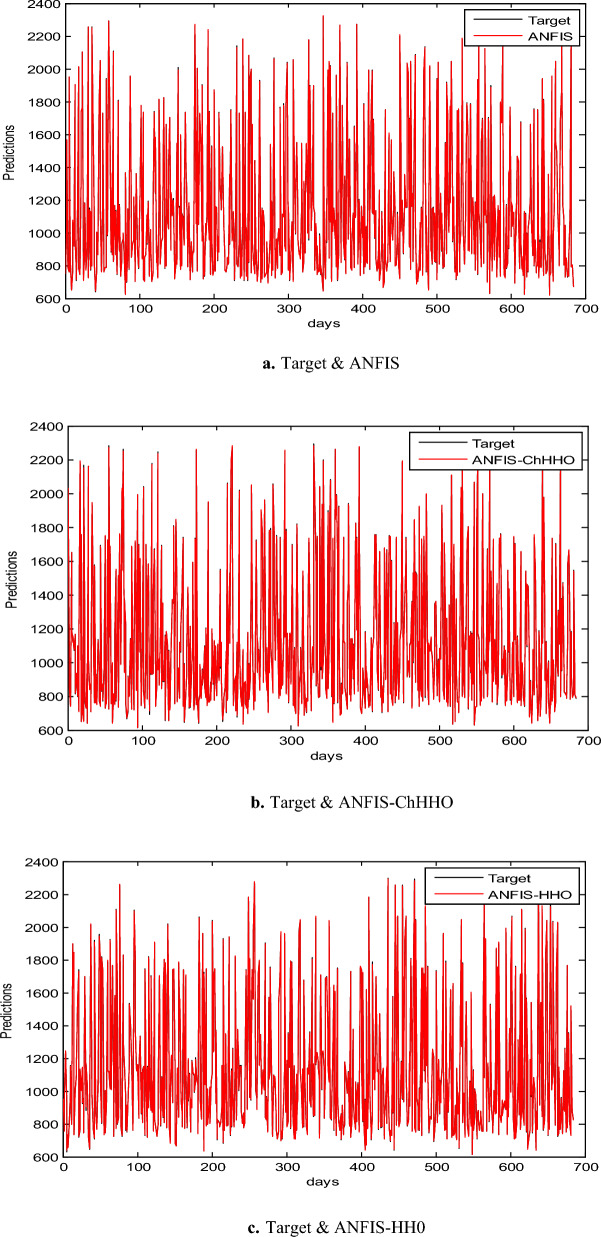

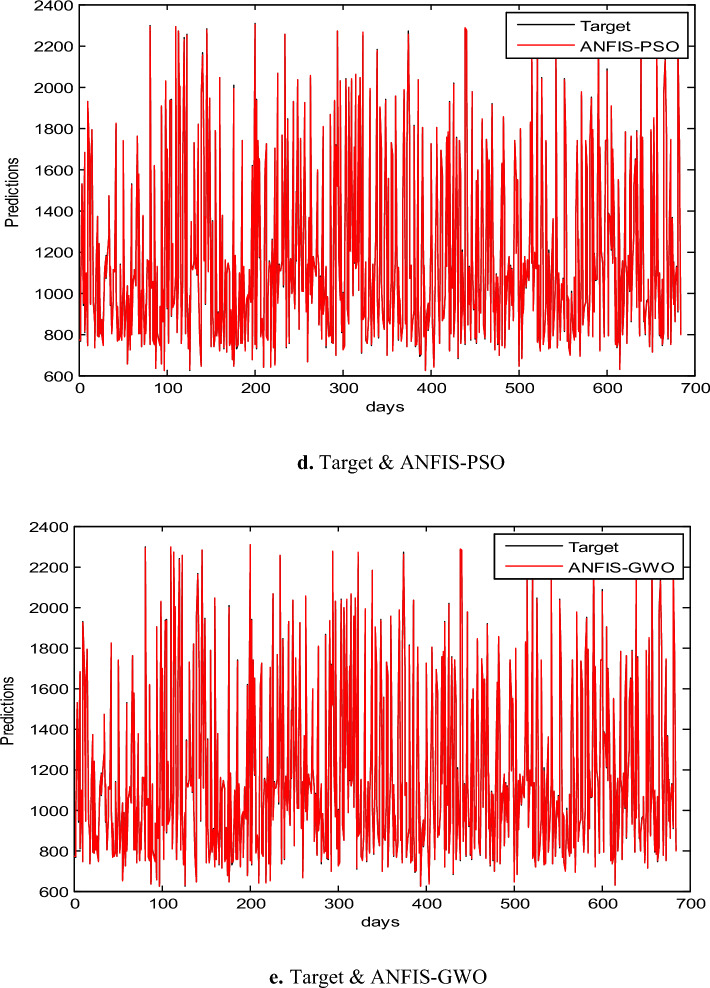
Prediction results of EGX-100 dataset.

**Fig. 8:**
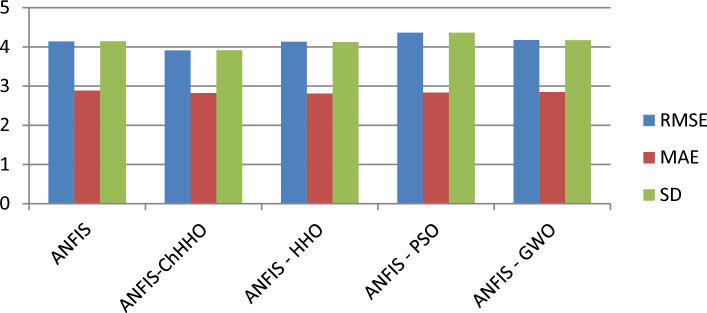
The results of metrics EGX-100 for testing the five models.

The proposed ANFIS-ChHHO approach is considered in this section using the EGX-100 dataset. Table 5 records the results of all models of the values, MAE, RMSE, SD and Theil’s U. As we can see, the ANFIS-ChHHO model got the best RMSE value, followed by the models ANFIS, ANFIS-GWO model, ANFIS-PSO, and ANFIS-HHO. Also, ANFIS- ChHHO obtained the best MAE value, while ANFIS came in second. After that, the ANFIS-GWO, ANFIS-PSO and ANFIS-HHO model. More, the ANFIS-ChHHO achieved the best Theil’s U value of 0.00161, and two models obtained the second rank ANFIS-GWO and ANFIS model, ANFIS-PSO model got the third rank, ANFIS-HHO came the fourth rank. Also, the ANFIS-ChHHO came in the first rank in the measured of SD, followed by ANFIS, ANFIS-GWO model, the ANFIS-PSO, and ANFIS-HHO. Moreover, Figs. 7a–e and 8 indicate the results of the all methods and the ANFIS-ChHHO for forecast of the next closing price day compared to other models is better and it is values that are closest to the original data.

The forecasting performance of ANFIS and its hybrid variants on the EGX-100 index is systematically evaluated across three key dimensions (Tables 9, 10, 11). Table 9 establishes ANFIS-ChHHO’s superior accuracy in absolute terms, achieving the lowest RMSE (3.8001) and MAE (2.7368), representing 2.8–11.3% improvements over other models. Table 10 reveals even more striking advantages in relative error metrics, where ANFIS-ChHHO demonstrates 42–74% reductions in MSRE (1.3892 × 10^−5^) and RMSPE (0.37272) compared to standard ANFIS. The comprehensive comparison in Table 11 consolidates these findings, showing ANFIS-ChHHO’s consistent top performance across all eight evaluation criteria, including SD (3.7873) and Theil’s U (0.00161). Notably, while ANFIS-HHO shows competitive absolute errors, its relative errors remain higher, and ANFIS-GWO emerges as the least accurate hybrid. These results collectively highlight ANFIS-ChHHO as the most reliable model for EGX-100 index prediction, particularly for applications requiring precise relative error control.

**Table 9:** Performance comparison of ANFIS and hybrid ANFIS models (ANFIS-ChHHO, ANFIS-HHO, ANFIS-PSO) in forecasting the EGX-100 index, evaluated using RMSE, MAE, SD, and Theil’s U metrics.

	RMSE	MAE	SD	Theil’s U
ANFIS	3.9093	2.7756	3.9121	0.00164
ANFIS–ChHHO	3.8001	2.7368	3.7873	0.00161
ANFIS–HHO	4.2834	2.9157	4.2865	0.0018
ANFIS–PSO	4.2405	2.8514	4.2285	0.00176
ANFIS–GWO	3.9973	2.781	3.9759	0.00164

Bold indicates the best results.

OR.

If used these metrics only.

**Table 10:** Comparative analysis of relative error metrics (MARE, MSRE, RMSRE, RMSPE) for ANFIS and its hybrid variants in predicting the EGX-100 index.

	MARE	MSRE	RMSRE	RMSPE
ANFIS	0.002621	1.7646e-5	0.0042008	0.42008
ANFIS-ChHHO	0.002595	1.3892e-5	0.0037272	0.37272
ANFIS-HHO	0.002719	1.9621e-5	0.0044296	0.44296
ANFIS-PSO	0.004643	4.4243e-5	0.0066515	0.66515
ANFIS-GWO	0.005294	5.3772e-5	0.0073329	0.73329

Bold indicates the best results.

OR.

If used these metrics additionally.

**Table 11:** Comprehensive performance evaluation of ANFIS and its hybrid variants (ANFIS-ChHHO, ANFIS-HHO, ANFIS-PSO, ANFIS-GWO) in forecasting the EGX-100 index, assessed through multiple accuracy metrics (RMSE, MAE, SD, Theil’s U, MARE, MSRE, RMSRE, RMSPE).

	ANFIS	ANFIS-ChHHO	ANFIS-HHO	ANFIS-PSO	ANFIS-GWO
RMSE	3.9093	3.8001	4.2834	4.2405	3.9973
MAE	2.7756	2.7368	2.9157	2.8514	2.781
SD	3.9121	3.7873	4.2865	4.2285	3.9759
Theil’s U	0.00164	0.00161	0.0018	0.00176	0.00164
MARE	0.002621	0.002595	0.002719	0.004643	0.005294
MSRE	1.7646e-5	1.3892e-5	1.9621e-5	4.4243e-5	5.3772e-5
RMSRE	0.0042008	0.0037272	0.0044296	0.0066515	0.0073329
RMSPE	0.42008	0.37272	0.44296	0.66515	0.73329

Bold indicates the best results.

The following figures, Fig. 7a–e, are the prediction results of EGX-100 dataset.

**Fig. 9:**
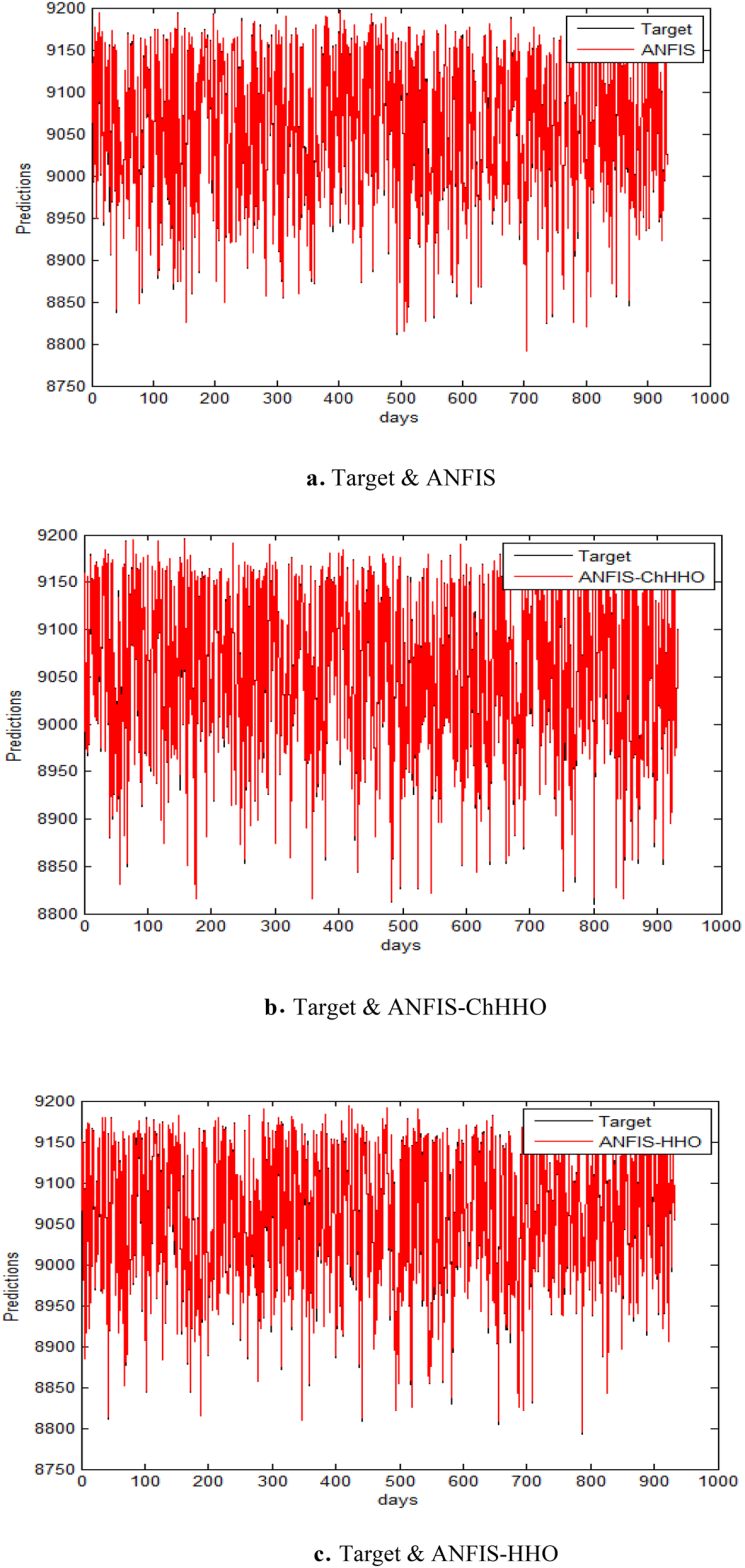

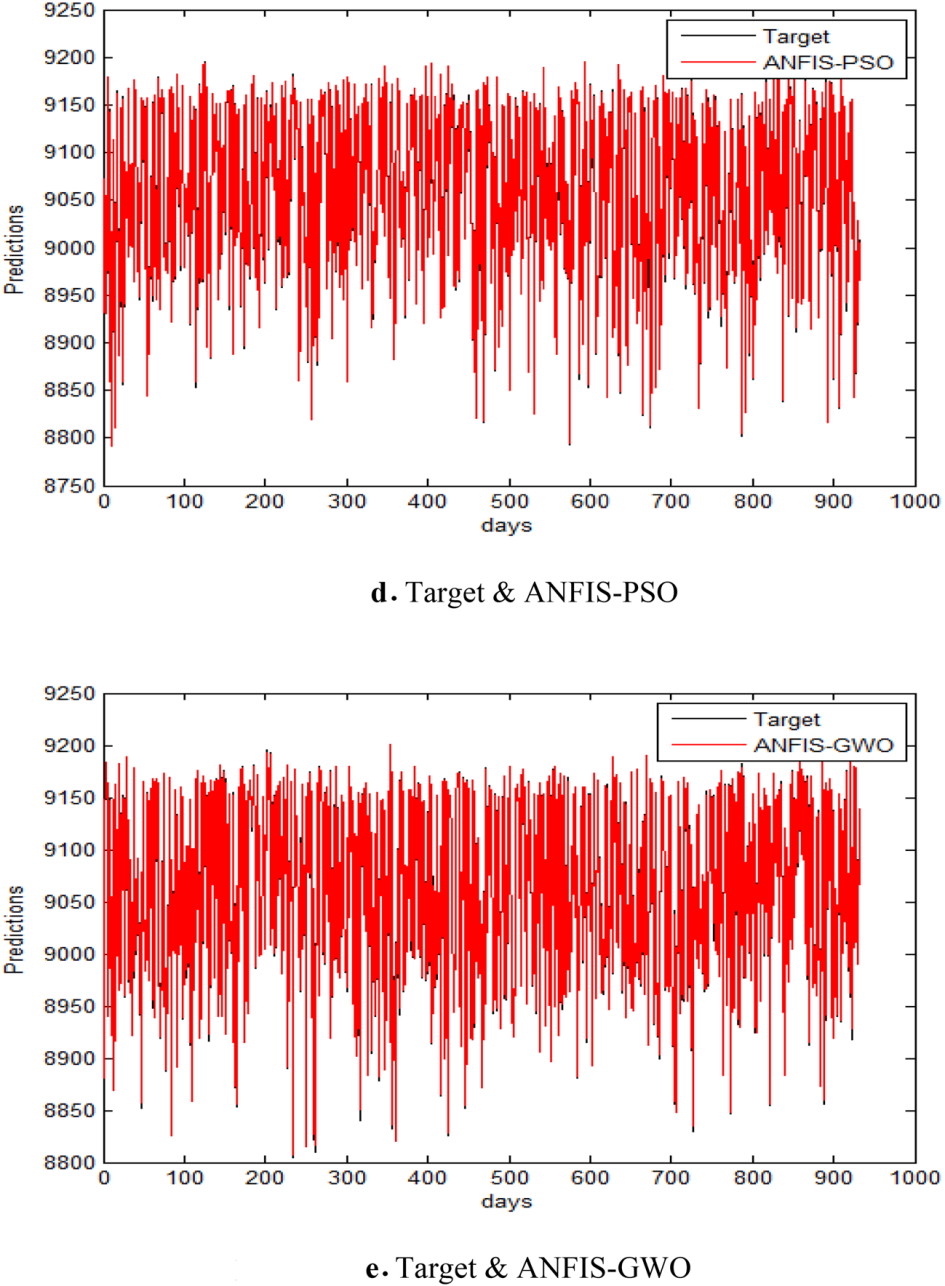
Prediction results of NASDAQ dataset.

**Fig. 10:**
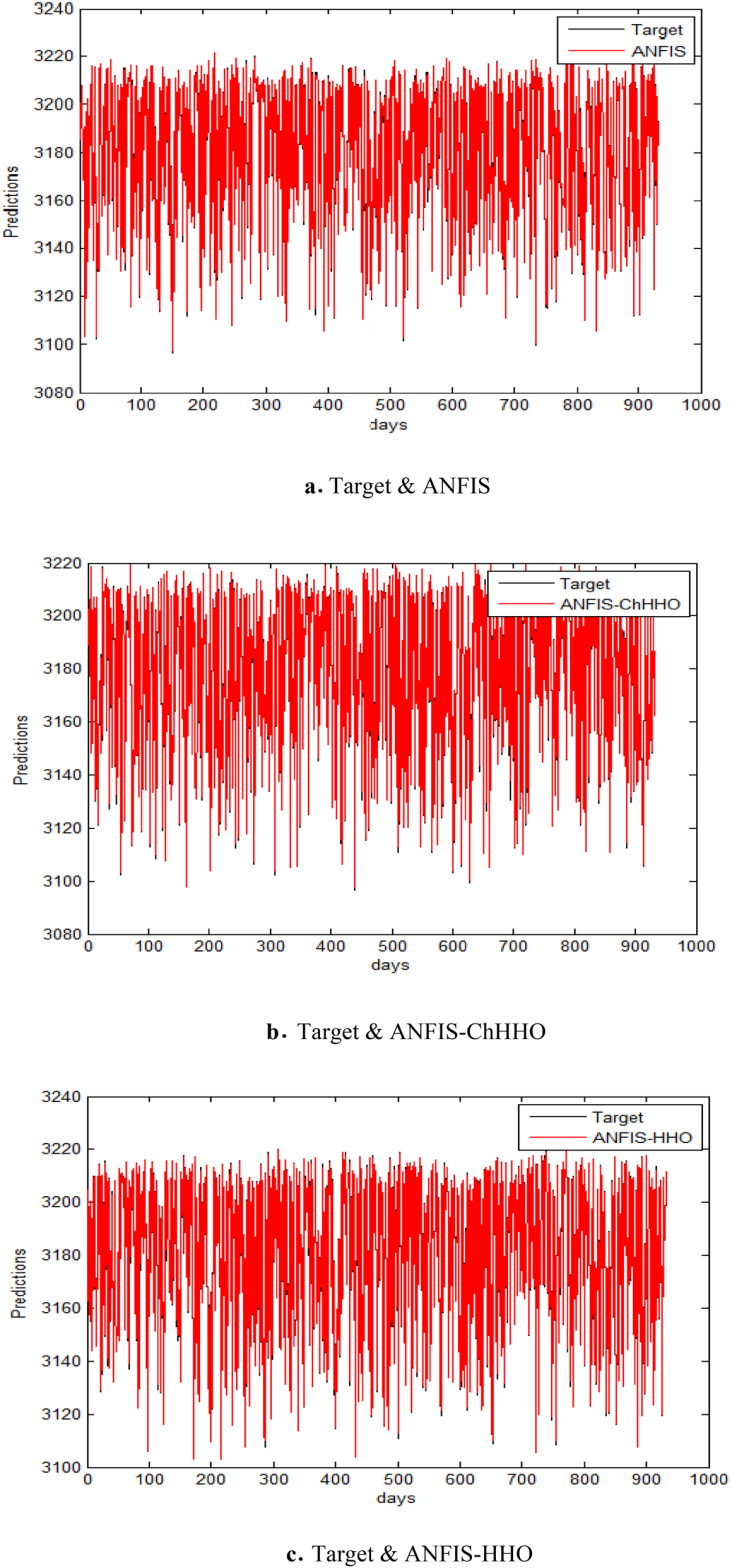

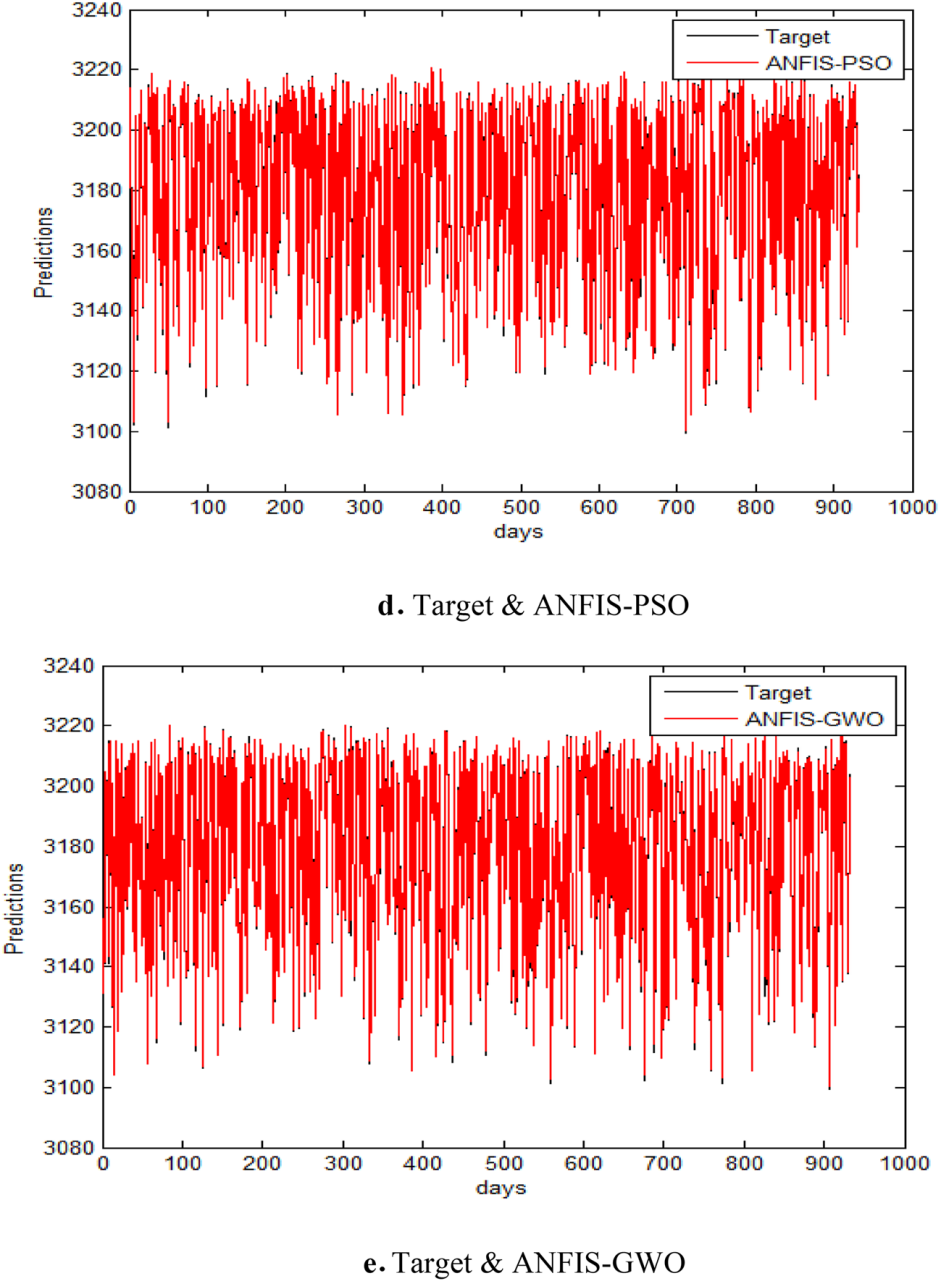
Prediction results of S&P 500 dataset.

In addition to the EGX datasets, we applied the model to the NASDAQ and S&P 500 indices. Figure 8 indicates the results of metrics EGX-100 for testing the five models. The results demonstrated consistent performance improvements, highlighting the model’s robustness across diverse financial environments as in Tables 12, 13, 14, 15, 16, and 17 and Figs. 9a–e and 10a–e.

**Table 12:** Comparative performance of ANFIS and hybrid ANFIS models in forecasting the NASDAQ index, evaluated through RMSE, MAE, SD, and Theil’s U metrics.

	RMSE	MAE	SD	Theil’s U
ANFIS	3.0418	1.84683	2.03684	0.0001872
ANFIS-ChHHO	2.1893	1.7183	2.05888	0.000142
ANFIS-HHO	3.0363	1.9148	3.0376	0.0001672
ANFIS-PSO	3.0288	1.8322	3.0242	0.000176
ANFIS-GWO	2.8068	1.8723	2.8082	0.000155

Bold indicates the best results.

**Table 13:** Relative error analysis of ANFIS and hybrid variants in NASDAQ index prediction.

	MARE	MSRE	RMSRE	RMSPE
ANFIS	0.0027003	1.2358 e-7	0.00033702	0.033702
ANFIS-ChHHO	0.00019011	8.2808e-8	0.00028776	0.028776
ANFIS – HHO	0.00021203	1.9621e-7	0.00033716	0.033716
ANFIS – PSO	0.00020295	1.1359e-7	0.00032602	0.033602
ANFIS-GWO	0.00020752	9.755e-6	0.00031233	0.031233

Bold indicates the best results.

**Table 14:** Comprehensive performance evaluation of ANFIS and hybrid variants in NASDAQ index forecasting, demonstrating ANFIS-ChHHO’s dominant performance across all metrics.

	ANFIS	ANFIS-ChHHO	ANFIS-HHO	ANFIS-PSO	ANFIS-GWO
RMSE	3.0418	2.1893	3.0363	3.0288	2.8068
MAE	1.84683	1.7183	1.9148	1.8322	1.8723
SD	2.03684	2.05888	3.0376	3.0242	2.8082
Theil’s U	0.0001872	0.000142	0.0001672	0.000176	0.000155
MARE	0.0027003	0.00019011	0.00021203	0.00020295	0.00020752
MSRE	1.2358e-7	8.2808e-8	1.9621e-7	1.1359e-7	9.755e-6
RMSRE	0.00033702	0.00028776	0.00033716	0.00032602	0.00031233
RMSPE	0.033702	0.028776	0.033716	0.032602	0.031233

Bold indicates the best results.

**Table 15:** Performance comparison of ANFIS and hybrid models in S&P 500 index forecasting, with ANFIS-ChHHO demonstrating superior predictive accuracy.

	RMSE	MAE	SD	Theil’s U
ANFIS	55.4932	39.4664	55.4447	0.0028796
ANFIS-ChHHO	38.1724	26.7329	38.0415	0.0019104
ANFIS-HHO	38.5157	28.9994	38.1743	0.0020951
ANFIS-PSO	41.1762	30.7713	41.1781	0.0021555
ANFIS-GWO	53.772	39.4551	53.6774	0.0027386

Bold indicates the best results.

**Table 16:** Relative error analysis of ANFIS-based models for S&P 500 index prediction, showing ANFIS-ChHHO’s superior performance with the lowest MARE (0.0032336), MSRE (2.0298 × 10^−5^), RMSRE (0.0045054), and RMSPE (0.45054).

	MARE	MSRE	RMSRE	RMSPE
ANFIS	0.0056688	5.3597e-5	0.0066028	0.66028
ANFIS-ChHHO	0.0032336	2.0298e-5	0.0045054	0.45054
ANFIS – HHO	0.003675	2.367e-5	0.0048651	0.48651
ANFIS – PSO	0.0037235	2.5016e-5	0.0050016	0.50016
ANFIS-GWO	0.0040022	5.1313e-5	0.0063016	0.63016

**Table 17:** Comprehensive performance evaluation of ANFIS and hybrid models for S&P 500 index forecasting, demonstrating ANFIS-ChHHO’s superior accuracy across all metrics.

	ANFIS	ANFIS-ChHHO	ANFIS-HHO	ANFIS-PSO	ANFIS-GWO
RMSE	55.4932	38.1724	38.5157	41.1762	53.772
MAE	39.4664	26.7329	28.9994	30.7713	39.4551
SD	55.4447	38.0415	38.1743	41.1781	53.6774
Theil’s U	0.0028796	0.0019104	0.0020951	0.0021555	0.0027386
MARE	0.0056688	0.0032336	0.003675	0.0037235	0.0040022
MSRE	5.3597e-5	2.0298e-5	2.367e-5	2.5016e-5	5.1313e-5
RMSRE	0.0066028	0.0045054	0.0048651	0.0050016	0.0063016
RMSPE	0.66028	0.45054	0.48651	0.50016	0.63016

Bold indicates the best results.

The datasets in both NASDAQ and S&P 500 from 8/11/2019 6:05:00 PM to 8/16/2019 4:10:00 AM and the data each 5 min. The number of rows 1214 . from (https://www.investing.com).

Results in Table 12 demonstrate ANFIS-ChHHO’s superior predictive accuracy with the lowest error values (RMSE = 2.1893, Theil’s U = 0.000142), while standard ANFIS and other hybrid variants show comparatively higher deviations. Notably, ANFIS-ChHHO achieves a 28% reduction in RMSE compared to baseline ANFIS.

Table 13 shows ANFIS-ChHHO’s exceptional performance with the lowest MARE (0.00019011), MSRE (8.2808e-8), RMSRE (0.00028776), and RMSPE (0.028776) – outperforming standard ANFIS by an order of magnitude in MARE and demonstrating 33–92% improvement across all metrics compared to other hybrid models.

In Table 14: The model achieves superior results with: lowest RMSE (2.1893), MAE (1.7183), Theil’s U (0.000142), MARE (0.00019011), MSRE (8.2808e-8), RMSRE (0.00028776), and RMSPE (0.028776) – showing 28–93% improvement over standard ANFIS and consistently outperforming other hybrid models in predictive accuracy and stability.

The following figures, Fig. 9a–e, indicate the prediction results of NASDAQ dataset.

The model in Table 15 achieves the lowest error metrics (RMSE = 38.1724, MAE = 26.7329, Theil’s U = 0.0019104), showing 31.2–32.3% improvement in RMSE and MAE over standard ANFIS, while maintaining the most stable predictions (SD = 38.0415). ANFIS-HHO shows comparable performance, whereas ANFIS-GWO performs closest to the baseline model.

OR

If used these metrics only 

As indicated in Table 16: the hybrid model demonstrates 29–62% improvement across all error metrics compared to standard ANFIS, with particularly strong gains in MSRE (62% reduction) and RMSPE (32% reduction). ANFIS-HHO and ANFIS-PSO show intermediate performance, while ANFIS-GWO remains closest to the baseline model’s results.

The optimal model in Table 17 achieves: lowest RMSE (38.1724, 31.2% improvement), MAE (26.7329, 32.3% reduction), Theil’s U (0.0019104), MARE (0.0032336), MSRE (2.0298 × 10^−5^), RMSRE (0.0045054), and RMSPE (0.45054), outperforming standard ANFIS by 29–62% across relative error measures. ANFIS-HHO shows comparable absolute errors but slightly higher relative errors, while ANFIS-GWO performs closest to the baseline, highlighting the effectiveness of the ChHHO optimization approach.

The following figures, Fig. 10a–e, are prediction results of S&P 500 dataset.

### Discussion

The sensitivity of the model to various input features was examined using a feature importance analysis. Results indicated that the open and high prices significantly influenced the prediction accuracy, while the low prices had a relatively lower impact. The computational complexity analysis demonstrated that the proposed ChHHO model significantly reduces convergence time compared to traditional methods, making it suitable for real-time forecasting.

The results from all datasets, including measures such as RMSE, MAE, SD, and Theil’s U, indicate that the proposed approach yields favorable outcomes across all utilized datasets. Compared to the classical ANFIS model, the proposed method attains the lowest error rate during the testing phase, demonstrating superior performance. Additionally, the various optimization techniques were assessed, including HHO, PSO, and GWO. The findings indicate that the proposed method surpassed all others regarding RMSE, MAE, SD, and Theil’s U. All tests in the study demonstrate the impact of ChHHO on optimizing the parameters of the ANFIS model.

In summary, the assessment experiments indicate that the implementation of the ChHHO substantially influences the efficacy of the classical ANFIS model. Furthermore, the chaotic mapping improved the efficacy of the basic HHO method, resulting in the ANFIS-ChHHO outperformed all other models in every measurable aspect.

Table 18 shows the predictive outcomes of several stock indices based on specific assessment measures through the current study and similar studies in literature.

**Table 18:** Comparison of similar studies in the literature.

Index	Model	MAE	RMSE	Theil’s U
DJI^17^	PGCSA-ELM	43.7481	–	0.00164
	CSA-ELM	98.3678		0.0030
	ELM	156.7445		0.0051
IXIC^17^	PGCSA-ELM	17.6458		0.0024
	CSA-ELM	26.9485		0.0028
	ELM	61.0248		0.0064
HSI^29^	IPSO-LSTM	277.472	367.745	–
S&P500^29^	RLSTM	12.0105	–	–
EGX-30	ANFIS-ChHHO	27.2965	38.2695	0.0019
EGX-70	ANFIS-ChHHO	1.5336	2.2414	0.0019
EGX-100 (current study)	ANFIS-ChHHO	2.7368	3.8001	0.00161

## Conclusions

In this paper, we propose an optimized Adaptive Neuro-Fuzzy Inference System (ANFIS) approach for stock market prediction. The classical ANFIS model has been enhanced using an improved Harris Hawks Optimization (HHO) algorithm to increase forecasting accuracy. The modified model, referred to as ANFIS-ChHHO, was evaluated using three datasets from the Egyptian Stock Exchange. To assess its performance, we compared the proposed model with the classical ANFIS model, as well as with other enhanced ANFIS variants, including ANFIS-HHO, ANFIS-PSO, and ANFIS-GWO.

The evaluation metrics used to compare the models were Root Mean Square Error (RMSE), Mean Absolute Error (MAE), Standard Deviation (SD), and Theil’s U. The results demonstrated that the ANFIS-ChHHO model significantly outperformed the classical ANFIS model and its enhanced counterparts, indicating superior forecasting precision.

Additionally, this study details the development of the HHO algorithm to improve its initial population by incorporating chaotic techniques, resulting in a more efficient search process and better convergence. The integration of the Levy distribution further enhances the HHO’s exploration capabilities when combined with chaotic mapping.

Future improvements to the ANFIS-ChHHO model could involve the incorporation of hybrid wavelet techniques to achieve even greater predictive accuracy. This potential enhancement could further optimize the model’s performance, especially in scenarios characterized by high market volatility.

Despite the promising results, the proposed model has certain limitations, including the computational overhead associated with chaotic mapping. Additionally, the model’s performance may vary with different stock datasets. Future research will explore integrating other hybrid optimization techniques, such as Genetic Algorithms, to further enhance accuracy and stability. Future research will explore the integration of deep learning techniques with chaotic optimization to further enhance predictive accuracy, especially in high-frequency trading scenarios.

## Data Availability

Data will be available on request by contacting with Dr. Zahraa Elsayed Mohamed via [zahraa_sd@yahoo.com] (mailto:zahraa_sd@yahoo.com).
